# Therapy-Induced Neutropenia and Poor Prognosis in Patients with Locally Advanced Esophageal Cancer Who Underwent Concurrent Chemoradiotherapy with Docetaxel, Cisplatin, and 5-Fluorouracil

**DOI:** 10.3390/cancers18010112

**Published:** 2025-12-29

**Authors:** Makoto Sakai, Nobuhiro Nakazawa, Kengo Kuriyama, Takuhisa Okada, Takuya Shiraishi, Yuji Kumakura, Akiharu Kimura, Akihiko Sano, Takehiko Yokobori, Ken Shirabe, Hiroshi Saeki

**Affiliations:** 1 Department of General Surgical Science, Graduate School of Medicine, Gunma University, Maebashi 371-8511, Japan; nakazawa75@gunma-u.ac.jp (N.N.); kkuriyama@gunma-u.ac.jp (K.K.); t.okd@gunma-u.ac.jp (T.O.); tashirai@gunma-u.ac.jp (T.S.); bokuha_kuma_kura@yahoo.co.jp (Y.K.); akimura@gunma-u.ac.jp (A.K.); ak_sano@gunma-u.ac.jp (A.S.); kshirabe@gunma-u.ac.jp (K.S.); h-saeki@gunma-u.ac.jp (H.S.); 2 Division of Gene Therapy Science, Initiative for Advanced Research (GIAR), Gunma University, Maebashi 371-8511, Japan; bori45@gunma-u.ac.jp

**Keywords:** esophageal cancer, chemoradiotherapy, overall survival, neutropenia

## Abstract

**Background:** The prognostic impact of therapy-induced neutropenia in patients receiving definitive chemoradiotherapy for locally advanced thoracic esophageal cancer (EC) remains inadequately characterized. This study aimed to evaluate the association between grade 3–4 neutropenia and survival outcomes following docetaxel–cisplatin–5-fluorouracil (DCF) combined with radiotherapy (DCF-RT). **Methods:** Fifty patients with locally advanced thoracic EC were included in this study. Chemotherapy consisted of intravenous docetaxel at 50 mg/m^2^ (day 1), CDDP at 60 mg/m^2^ (day 1), and 5-FU at 600 mg/m^2^ (days 1 to 4), administered every four weeks for two cycles in combination with radiotherapy (60 Gy in 30 fractions). Toxicities were assessed using the Common Terminology Criteria for Adverse Events. Overall survival (OS), progression-free survival (PFS), locoregional control and distant metastasis-free survival were compared by neutropenia grade. **Results:** Grade 3–4 neutropenia occurred in 80% (95% CI: 66.3–90.0) of patients. The OS rate was significantly lower in those with grade 3–4 neutropenia than in those with grade 0–2 (*p* = 0.006). Multivariate analysis identified grade 3–4 neutropenia (HR 3.77; 95% CI: 1.35–10.56) and complete response (CR) (HR 0.47; 95% CI: 0.25–0.87) as independent prognostic factors for OS among patients who received definitive CRT. Among 38 patients with recurrence or residual disease, those with grade 3–4 neutropenia exhibited significantly greater reductions in lymphocyte counts at recurrence versus pretreatment (p = 0.012) compared with those with grade 0–2 neutropenia. **Conclusions:** Therapy-induced neutropenia is an independent prognostic factor for OS in locally advanced thoracic EC patients undergoing definitive DCF-RT. It may also serve as a predictor of insufficient lymphocyte recovery following chemoradiation.

## Simple Summary

Therapy-induced neutropenia is a common complication that occurs during definitive chemoradiotherapy for locally advanced esophageal cancer, but its prognostic significance is still unclear. In this study, we found that patients who developed severe neutropenia (Grade 3–4) during treatment had significantly worse overall survival compared to those with mild or no neutropenia. Notably, severe neutropenia was linked to greater reductions in lymphocyte counts at the time of cancer recurrence, indicating an impaired ability of the immune system to recover. These findings suggest that therapy-induced neutropenia not only reflects treatment toxicity but may also predict insufficient lymphocyte recovery and compromised antitumor immunity. Our results emphasize the need for further prospective studies to better understand the clinical and immunological consequences of myelosuppression in multimodal esophageal cancer therapy.

## 1. Introduction

Esophageal cancer (EC) is the seventh leading cause of death from cancer and the 11th most common cancer worldwide [1]. The 10-year net survival rate of EC diagnosed in 2011 was approximately 30% in Japan [2]. Concurrent chemoradiotherapy (CRT) is the standard therapy for unresectable esophageal cancer and is also recognized as an option for resectable cancer. Definitive CRT is a treatment option for patients who refuse or cannot tolerate surgery [3,4]. Currently, the most commonly used regimen for esophageal cancer with concurrent radiation therapy is a combination of cisplatin and 5-fluorouracil (CF) [5,6]; however, the effectiveness of this regimen remains insufficient in terms of local control and survival rates [6,7,8]. We previously reported that docetaxel, cisplatin, and 5-fluorouracil as combination chemoradiotherapy (DCF-RT) led to a high complete response (CR) rate and favorable prognosis compared with CF-RT for advanced esophageal cancer [9]. Therapy-induced myelosuppression has been reported to be a prognostic factor in several tumors [10,11,12,13,14,15]. The clinical significance of treatment-induced myelosuppression can be considered from two perspectives. One perspective is that myelosuppression may act as a negative prognostic factor because it can lead to treatment interruptions or dose reductions, thereby limiting the planned therapy. The other perspective is that myelosuppression may serve as a surrogate marker of antitumor efficacy, reflecting that treatment was delivered with appropriate dose intensity. Severe neutropenia during treatment may therefore integrate information on both toxicity and treatment intensity. However, the precise implications of therapy-induced myelosuppression for patient outcomes remain largely unclear and require further investigation. As definitive DCF-RT is a highly myelotoxic treatment, understanding the prognostic significance of therapy-induced myelosuppression is crucial not only for risk stratification, but also for optimizing treatment delivery and balancing efficacy with tolerability. This study aimed to explore the effect of therapy-induced neutropenia on clinical outcomes of DCF-RT for locally advanced thoracic EC.

## 2. Materials and Methods

### 2.1. Patients

This retrospective study was approved by the Ethics Committee of the Graduate School of Medicine, Gunma University (Protocol number: HS2019-319). Informed consent was obtained by an opt-out on our website. We typically administer DCF-RT to patients with unresectable EC or those who refuse or cannot tolerate surgery, unless they have severe myelosuppression or renal dysfunction. Between July 2008 and December 2018, 61 patients underwent DCF-RT for unresectable locally advanced thoracic EC at our institute. Inclusion criteria were (1) histologically confirmed thoracic esophageal squamous cell carcinoma; (2) unresectable, locally advanced disease (clinical T4 and/or clinical N0-3, M0 or M1 limited to supraclavicular lymph nodes); (3) no prior treatment for esophageal cancer; (4) planned definitive chemoradiotherapy with DCF and 60 Gy/30 fractions of 3-dimensional conformal radiotherapy (3D-conformal RT). Exclusion criteria were distant organ metastasis, synchronous cancer, pretreatment chemotherapy, and those that received different radiation protocols (such as patients treated with intensity-modulated radiation therapy boost after the initial 40 Gy). Of the 61 patients, 50 were enrolled. Hospital patient records were reviewed for tumor characteristics and patient outcomes. Tumor stage and disease grade were classified according to the 8th edition of the TNM classification of the International Union Against Cancer [16]. Tumor stage was determined conventionally using computed tomography (CT) and positron emission tomography (PET)-CT of the neck, chest, and abdomen, as well as endoscopic ultrasonography, endoscopy, and esophagography.

### 2.2. Treatment

Patients received concurrent radiotherapy and chemotherapy for six weeks after diagnostic procedures. Chemotherapy consisted of DCF (intravenous docetaxel [50 mg/m^2^] on day 1, intravenous CDDP [60 mg/m^2^] on day 1, and intravenous 5-FU [600 mg/m^2^] on days 1 to 4), administered every four weeks for two cycles. Patients were irradiated with 1.8 to 2.0 Gy/fraction using 10-MV photons from a linear accelerator. The initial 40 Gy was delivered to the initial radiation field involved the primary tumor and lymph node region with metastasis using anterior–posterior opposed fields. The boost dose of 20 Gy was delivered to the shrunken primary tumor and metastatic lymph nodes using oblique parallel opposed fields to avoid the spinal cord [9]. The irradiated doses were prescribed to the plan isocenter in principle. Dose constraints for thoracic vertebral/sternal bone marrow were not applied. After receiving 30–40 Gy of irradiation, patients deemed resectable were considered for conversion to esophagectomy 3 to 4 weeks later (conversion surgery). Some patients received CF-based consolidation chemotherapy after completing radiation if they showed no disease progression and had adequate organ function. Toxicities were assessed using the Common Terminology Criteria for Adverse Events v4.0. Blood tests, including complete blood counts with differential, were routinely performed one week after the start of each chemotherapy cycle. Subsequent laboratory evaluations were conducted based on clinical symptoms at the treating physician’s discretion. Patients were categorized into two groups: those with Grade 0–2 neutropenia and those with Grade 3–4 neutropenia, based on the most severe occurrence during CRT.

### 2.3. Response Evaluation

Standard clinical measurements and radiological examinations were used to assess the tumor response according to RECIST criteria. The treatment response of the primary lesion (non-target lesion) was evaluated according to the Japanese Classification of Esophageal Cancer, 11th edition [17]. Details of response evaluation were previously described [9].

### 2.4. Follow-Up

Patients were evaluated every three months after completing treatment for the first two years and then every six months thereafter. CR was confirmed through endoscopy, biopsy, CT, and PET-CT.

### 2.5. Statistical Analysis

Locoregional failure was defined as the persistence or recurrence of the primary tumor or regional lymph nodes. Distant failure was defined as metastasis to any site beyond the primary tumor and regional lymph nodes. For locoregional and distant failure, cumulative incidence functions were estimated treating death as a competing event, and differences between groups were evaluated using Gray’s test. Overall survival (OS) was calculated from the start of treatment to the last follow-up or death. Progression-free survival (PFS) was defined as the time from the start of treatment to the first occurrence of disease progression at any site, recurrence, or death from any cause. Locoregional control survival (LCS) was defined as the time from the start of treatment to the first occurrence of locoregional recurrence, including recurrence at the primary tumor site or regional lymph nodes, or death from any cause. Distant metastasis-free survival (DMFS) was defined as the time from the start of treatment to the development of distant metastasis outside the locoregional area, or death from any cause. Kaplan–Meier curves were generated for OS, PFS, LCS, and DMFS. Univariate and multivariate survival analyses were performed using Cox proportional hazards regression model. Variables were selected using a stepwise selection to minimize the Akaike information criterion [18] and were then included in a multivariate Cox proportional hazards model. Because some patients underwent additional treatments during or following CRT, including additional surgery (conversion or salvage surgery) and consolidation chemotherapy, these post-treatment interventions were modeled as time-dependent covariates in the multivariate Cox model. A probability value of <0.05 was considered significant. All statistical analyses were performed using EZR software (version 1.68) [19].

## 3. Results

### 3.1. Patient Characteristics and Neutropenia

Grade 3–4 neutropenia was confirmed in 40 patients (80.0%) (95% confidence interval [CI], 66.3–90.0). The median follow-up period was 21.1 months (range 3.2–109.7). Table 1 shows the association between therapy-induced neutropenia and baseline patient characteristics. There were no significant differences in patient characteristics according to neutropenia grade.

### 3.2. Treatment-Related Factors and Neutropenia

Table 2 shows the association between therapy-induced neutropenia and treatment-related factors. The overall CR rate of patients underwent definitive CRT was 40.0% (95% confidence interval [CI], 24.9–56.7). The average relative dose intensity (ARDI) in the first cycle of DCF was 99.4%, and that in the second cycle was 71.1%. There were no significant differences in ARDI between neutropenia grades (*p* for 1st cycle = 0.227, p for 2nd cycle = 0.800). Grade 3–4 neutropenia was significantly associated with Grade 3–4 leukopenia (*p* = 0.010) but not with Grade 3–4 lymphocytopenia (*p* = 1.000). Consolidation chemotherapy was administered to 29 patients underwent definitive CRT (67.5% (95% CI: 50.9–81.4)). Conversion surgery was performed in 10 patients (20.0% (95% CI: 10.0–33.7)). No significant differences were found in the rate of consolidation chemotherapy (*p* = 0.236) and conversion surgery (*p* = 1.000) according to neutropenia grade. Per-agent RDI for the second chemotherapy cycle was analyzed in patients receiving definitive CRT, excluding conversion surgery cases (Supplementary Table S1). The mean RDIs for docetaxel, cisplatin, and 5-FU were 72.7%, 71.3%, and 71.6%, respectively, in patients with grade 3–4 neutropenia, compared with 68.0%, 68.0%, and 68.0% in patients with grade 0–2 neutropenia, with no statistically significant differences. Chemotherapy dose reductions or omissions, G-CSF administration, and antibiotic use did not differ significantly between patients with grade 0–2 and grade 3–4 neutropenia. G-CSF was used only for therapeutic purposes, and antibiotics were administered as clinically indicated, with no prophylactic use.

### 3.3. Failure Pattern and Salvage Treatment

Table 3 shows the initial failure patterns. Thirty-eight patients (76% (95% CI: 61.8–86.9)) had recurrence or residual disease at the time of analysis. Among them, 26 patients (52.0% (95% CI: 37.4–66.3)) received treatment for recurrence or residual disease, and four patients (8.0% (95% CI: 2.2–19.2)) underwent salvage surgery. There were no significant differences in initial failure pattern and salvage treatment between the neutropenia grades (*p* = 0.681).

**Figure 1:**
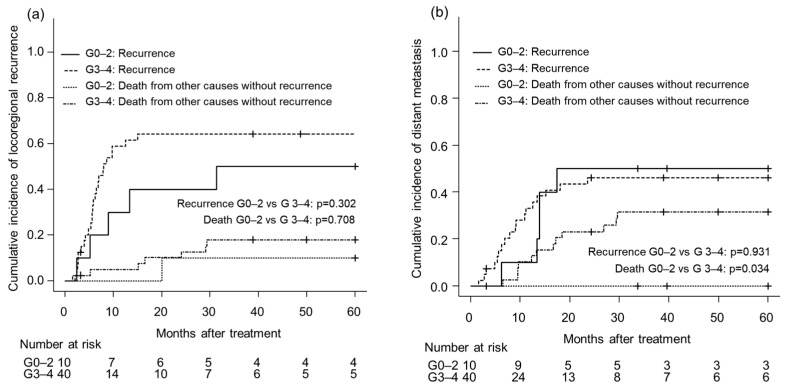
Cumulative incidence of (**a**) locoregional and (**b**) distant metastasis according to neutropenia grade.

Time-to-event analyses specific to failure patterns were conducted using competing-risk methods. For locoregional recurrence, no significant difference in cumulative incidence was observed between the G0–2 and G3–4 neutropenia groups (*p* = 0.302) (Figure 1a). Similarly, the cumulative incidence of distant metastasis was comparable between the two groups (*p* = 0.931) (Figure 1b). However, death without prior distant metastasis occurred slightly more frequently in the G3–4 group (*p* = 0.034), although the number of events was limited.

### 3.4. Survival Analysis

**Figure 2:**
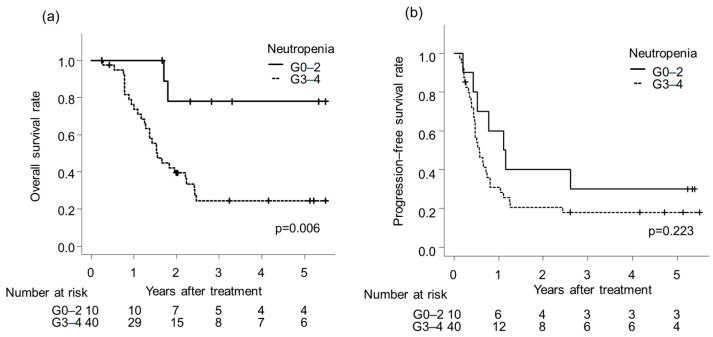
Kaplan–Meier curves for (**a**) overall survival and (**b**) progression-free survival rate according to neutropenia grade.

The OS rate was significantly lower for patients with Grade 3–4 neutropenia than those with Grade 0–2 neutropenia (*p* = 0.006; Figure 2a). The PFS rate did not significantly differ between the grade 0–2 and grade 3–4 neutropenia groups (*p* = 0.223; Figure 2b). Similarly, LCS (Supplementary Figure S1a) and DMFS (Supplementary Figure S1b) rates showed no significant difference across neutropenia grades (p for LCS = 0.226, p for DMFS = 0.692).

**Figure 3:**
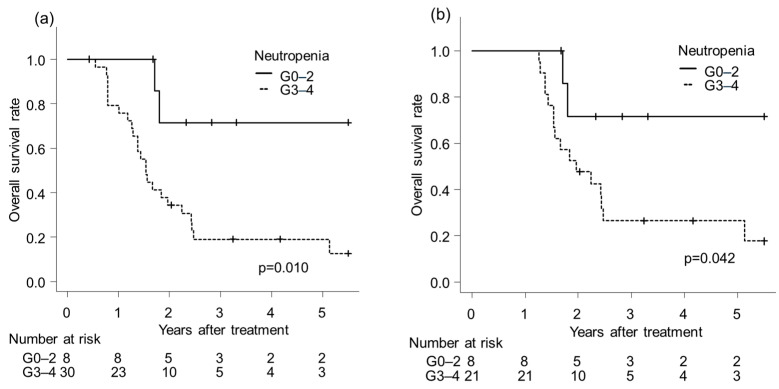
Landmark analysis of overall survival in patients with a minimum survival of (**a**) 5 months and (**b**) 15 months, stratified by neutropenia grade.

To account for potential immortal-time bias related to post-CRT interventions, we performed landmark analyses. For conversion surgery, the interval from treatment initiation to surgery ranged from 2.04 to 2.64 months. As no deaths occurred among patients who did not undergo conversion surgery during this period, a 2-month landmark analysis was not performed. For consolidation chemotherapy, after excluding patients who underwent conversion surgery, the latest initiation was 4.8 months after treatment start, and a 5-month landmark analysis was conducted (Figure 3a). For salvage surgery, the latest initiation among patients without conversion surgery was 15 months after treatment start, and a 15-month landmark analysis was performed (Figure 3b). In all landmark analyses, patients with G0–2 neutropenia demonstrated significantly better survival than those with G3–4 neutropenia (*p* = 0.010 for consolidation chemotherapy; *p* = 0.041 for salvage surgery).

In the univariate and multivariate analyses of OS among all patients, CR or non-CR was not included as a covariate because it was not possible to accurately classify the definitive CRT response in patients who underwent conversion surgery during CRT. In this multivariate analysis, grade 3–4 neutropenia was an independent prognostic factor for OS (HR 3.76 (95% CI: 1.36–10.4)) (Table 4). Then, to prevent misclassification of their response status, we conducted univariate and multivariate analyses of OS among patients who received definitive CRT, excluding those who underwent conversion surgery. In this specific group, the CR versus non-CR status after completing CRT was included as a binary covariate in a Cox model to evaluate the prognostic significance of the definitive CRT response. In this limited analysis of patients who received definitive CRT, multivariate analysis demonstrated that CR (HR 0.47 (95% CI: 0.25–0.87)) and grade 3–4 neutropenia (HR 3.77 (95% CI: 1.35–10.56)) were independent prognostic factors for OS (Table 5).

### 3.5. Lymphocyte Counts and Neutropenia in Recurrent or Residual Disease

Among the 38 patients with recurrence or residual disease, no significant differences were found between therapy-induced neutropenia and pretreatment lymphocyte count (*p* = 0.284), nor between lymphocyte count at the time of recurrence or residual disease (*p* = 0.147). However, Grade 3–4 neutropenia was associated with a significantly lower time of recurrence (residual) to pretreatment lymphocyte ratio (calculated as the lymphocyte count at the time of recurrence or residual disease divided by the pretreatment lymphocyte count) (*p* = 0.012; Table 6). Lymphocyte counts at the time of recurrence or residual disease were significantly more reduced from pretreatment levels in patients with Grade 3–4 neutropenia than those with Grade 0–2 neutropenia during chemoradiation.

## 4. Discussion

The most important finding of the present study was that therapy-induced neutropenia was an independent prognostic factor for OS in locally advanced thoracic EC patients who underwent definitive DCF-RT. Therapy-induced myelosuppression has been reported to be a poor prognostic factor in EC [10,11,12,13]. Miyoshi et al. reported that the toxic grade for leukopenia was a significant prognostic factor in patients with T4 esophageal cancer who underwent CRT followed by curative resection [12]. Ohira et al. reported that CRT-induced leukopenia has a negative impact on the prognosis of patients with locally advanced esophageal squamous cell carcinoma (ESCC) followed by surgery [10]. Li et al. also reported that grade 4 lymphopenia during neoadjuvant CRT was significantly associated with a lower pCR rate and a higher recurrence risk in ESCC patients [13]. Although these previous studies were in the neoadjuvant setting, our study supports their findings and suggests the prognostic significance of chemoradiation-induced myelosuppression in EC patients, even in a definitive setting.

In contrast to the concept that neutropenia may reflect adequate chemotherapy exposure and improved tumor control [14,15,20], our data do not support this beneficial interpretation. ARDI, per-agent RDI, CR rate, and post-CRT salvage or consolidation treatments were not significantly different across neutropenia grades. Thus, treatment delivery was maintained regardless of neutrophil toxicity, indicating that reduced dose intensity was unlikely to account for the inferior survival associated with severe neutropenia. These findings suggest that therapy-induced neutropenia represents a biological vulnerability rather than a surrogate for chemotherapy sufficiency.

Several mechanisms may explain the adverse impact of severe neutropenia on survival. Neutrophils have been reported to have several antitumor effects. Neutrophils stimulated by interferon release tumor necrosis factor-related apoptosis-inducing ligand, which promotes apoptosis in tumor cells [21]. Tumor-entrained neutrophils can inhibit tumor metastasis by inducing tumor cell death via hydrogen peroxide [22]. Furthermore, intratumoral neutrophil infiltration predicts better OS in ESCC patients [23]. In this context, the drug’s toxic effect on neutrophils and the resulting decrease in neutrophil count may lead to an unfavorable treatment response and worse tumor prognosis. The antitumor aspects of neutrophils may be part of the mechanism underlying the association between chemoradiotherapy-induced neutropenia and poor prognosis in EC patients.

Additionally, impaired systemic immunity may play a major role. Lymphocytes play a critical role in promoting systemic antitumor responses. In lung cancer patients, the persistence of lymphopenia 3 months after CRT has been reported to be associated with worse OS and progression-free survival [24]. In EC patients, an insufficient level of lymphocyte recovery after definitive CRT has been reported to be significantly associated with worse OS and local recurrence-free survival [25]. In our study, most patients (96%) presented Grade 3–4 lymphocytopenia during CRT; thus, stratifying the patients by lymphocytopenia grade was not performed. However, severe neutropenia was associated with a significantly lower lymphocyte-recovery ratio at recurrence, despite similar pretreatment lymphocyte levels. This suggests that therapy-induced neutropenia may serve as a clinical surrogate for sustained immune suppression. Considering that absolute or relative lymphocyte counts predict therapeutic benefit from ICIs in patients receiving immunotherapy, such as nivolumab for melanoma or recurrent esophageal cancer [26,27], CRT-related myelosuppression could negatively influence outcomes in patients subsequently treated with immunotherapy. Although speculative due to the lack of ICI exposure in this cohort, this relationship warrants future investigation.

Interestingly, there were no significant differences in PFS, LCS, and DMFS among the different grades of neutropenia. However, OS was worse in patients with severe neutropenia. Competing-risk analysis showed a slightly higher rate of death without prior distant metastasis in the grade 3–4 group. This suggests that compromised systemic immunity, rather than accelerated metastatic failure, may contribute to the reduced survival observed in these patients. This survival pattern further reinforces the idea that therapy-induced neutropenia indicates a vulnerability in host-tumor interactions, which is independent of tumor control.

This study has several limitations. First, its retrospective design and the fact that it was conducted at a single institution with a relatively small sample size may have introduced selection bias and limited statistical power. Therefore, the generalizability of these results requires confirmation in larger multicenter prospective cohorts. Second, although the development of grade 3–4 neutropenia was clearly documented, the duration of severe neutropenia could not be evaluated. Blood tests after the first week of chemotherapy were not performed on a fixed schedule, and in some cases, differential counts were unavailable due to clinical constraints. Because not only the occurrence but also the duration and cumulative burden of myelotoxicity may influence treatment tolerance, immune recovery, and survival outcomes, the inability to accurately quantify exposure-time limits interpretation of the biological mechanism. Future prospective studies incorporating standardized serial blood sampling are needed. Third, radiotherapy dose–volume parameters for thoracic bone marrow (including vertebral bodies and the sternum) could not be assessed, as bone marrow was not routinely contoured during treatment planning during the study period. Considering recent findings that thoracic bone marrow irradiation may be associated with hematologic toxicity during CRT for esophageal cancer [28], the inability to evaluate marrow dose constraints represents an important limitation that future studies should address. Fourth, chemotherapy dose reductions and modifications were performed at the discretion of treating physicians. Although no significant difference in dose intensity was observed between neutropenia groups, residual confounding from individualized treatment decisions cannot be entirely excluded. Finally, while our findings suggest that profound CRT-induced myelosuppression may lead to inadequate lymphocyte recovery and potentially influence antitumor immunity, its impact on subsequent immunotherapy outcomes remains speculative. Because our study cohort predates the widespread introduction of ICIs, future prospective studies integrating longitudinal hematologic monitoring and ICI treatment responses will be necessary to validate this hypothesis.

## 5. Conclusions

In conclusion, therapy-induced neutropenia has been identified as an independent prognostic factor for OS in patients receiving definitive DCF-RT. Severe neutropenia may indicate inadequate lymphocyte recovery and a weakened antitumor immune response. Understanding the relationship between treatment-related myelosuppression and the host’s immune competence will be essential for optimizing future therapeutic strategies for EC.

## Figures and Tables

**Table 1:** Association between therapy-induced neutropenia and baseline patient characteristics.

Characteristics	All Patients	Neutropenia		
		Grade 0–2	Grade 3–4	*p*-Value
	(*n* = 50)	(*n* = 10)	(*n* = 40)	
Age, years (mean [SD])	65.0 (8.6)	66.5 (8.3)	67.2 (7.5)	0.791
Sex, No. (%)				
Female	5 (10.0)	1 (10.0)	4 (10.0)	1
Male	45 (90.0)	9 (90.0)	36 (90.0)	
Location of tumor, No. (%)				
Ut	12 (24.0)	3 (30.0)	9 (22.5)	0.161
Mt	34 (68.0)	5 (50.0)	29 (72.5)	
Lt	4 (8.0)	2 (20.0)	2 (5.0)	
Lymph node metastasis, No. (%)				
cN0	4 (8.0)	0 (0.0)	4 (10.0)	0.772
cN1	30 (60.0)	6 (60.0)	24 (60.0)	
cN2	14 (28.0)	4 (40.0)	10 (25.0)	
cN3	2 (4.0)	0 (0.0)	2 (5.0)	
M1, No. (%)				
cM0	35 (70.0)	6 (60.0)	29 (72.5)	0.462
* cM1 (LYM)	15 (30.0)	4 (40.0)	11 (27.5)	
Pretreatment neutrophil count (cells/μL, mean [sd])	5481 (2799)	4931 (1616)	5618 (3024)	0.493
Pretreatment lymphocyte count (cells/μL, mean [sd])	1332 (475)	1194 (488)	1367 (472)	0.308

* Supraclavicular lymph node metastasis.

**Table 2:** Association between therapy-induced neutropenia and treatment-related factors.

Characteristics	All Patients	Neutropenia		
		Grade 0–2	Grade 3–4	*p*-Value
	(*n* = 50)	(*n* = 10)	(*n* = 40)	
* Treatment Response, No. (%)				
Non-CR	24 (60.0)	4 (50.0)	20 (62.5)	0.690
CR	16 (40.0)	4 (50.0)	12 (37.5)	
Average relative dose intensity (1st cycle) (mean [sd])	99.4 (4.2)	98.0 (6.3)	99.8 (3.5)	0.227
* Average relative dose intensity (2nd cycle) (mean [sd])	71.1 (37.7)	68.0 (43.9)	71.8 (36.7)	0.800
Leukopenia, No. (%)				
Grade 0–2	9 (18.0)	5 (50.0)	4 (10.0)	0.010
Grade 3–4	41 (82.0)	5 (50.0)	36 (90.0)	
Lymphocytopenia, No. (%)				
Grade 0–2	2 (4.0)	0	2 (5.0)	1
Grade 3–4	48 (96.0)	10 (100.0)	38 (95.0)	
* Consolidation chemotherapy, No. (%)				
−	13 (32.5)	1 (12.5)	12 (37.5)	0.236
+	27 (67.5)	7 (87.5)	20 (62.5)	
Conversion Surgery, No. (%)				
−	40 (80.0)	8 (80.0)	32 (80.0)	1
+	10 (20.0)	2 (20.0)	8 (20.0)	

* Analysis in patients without conversion surgery.

**Table 3:** Initial failure pattern and treatment.

Characteristics	All Patients	Neutropenia		
		Grade 0–2	Grade 3–4	*p*-Value
	(*n* = 50)	(*n* = 10)	(*n* = 40)	
No recurrence or residual disease, No. (%)	12 (24.0)	3 (30.0)	9 (22.5)	0.686
Locoregional, No. (%)	23 (60.5)	5 (71.4)	18 (58.1)	0.681
Distant, No. (%)	15 (39.5)	2 (28.6)	13 (41.9)	0.681
Treatment for recurrence or residual disease, No. (%)				
−	24 (48.0)	5 (50.0)	19 (47.5)	1
+	26 (52.0)	5 (50.0)	21 (52.5)	
Salvage Surgery, No. (%)				
−	46 (92.0)	8 (80.0)	38 (95.0)	0.174
+	4 (8.0)	2 (20.0)	2 (5.0)	

**Table 4:** Univariate and multivariate analyses for overall survival for all patients.

	Univariate		Multivariate	
Characteristics	HR (95% CI)	*p*-Value	HR (95% CI)	*p*-Value
Age, years	1.00 (0.95–1.05)	0.92		
Sex				
Female (reference)	1	0.600		
Male	1.37 (0.41–4.54)			
Lymph node metastasis				
Negative (reference)	1	0.500	1	0.210
Positive	1.64 (0.39–6.87)		2.52 (0.59–10.65)	
* Additional surgery (Conversion and salvage)				
− (reference)	1	0.061	1	0.070
+	0.42 (0.17–1.04)		0.44 (0.18–1.07)	
Neutropenia				
Grade 0–2 (reference)	1	0.013	1	0.011
Grade 3–4	6.17 (1.47–25.94)		3.76 (1.36–10.40)	

* Modeled as time-dependent covariates.

**Table 5:** Univariate and multivariate analyses for overall survival for patients underwent definitive CRT.

	Univariate		Multivariate	
Characteristics	HR (95% CI)	*p*-Value	HR (95% CI)	*p*-Value
Age, years	1.01 (0.96–1.06)	0.64	1.82 (0.76–4.32)	0.180
Sex				
Female (reference)	1	0.420		
Male	1.43 (0.61–3.35)			
Lymph node metastasis				
Negative (reference)	1	0.270	1	0.074
Positive	3.07 (0.41–22.79)		6.44 (0.83–49.69)	
Treatment Response				
Non-CR (reference)	1	0.015	1	0.016
CR	0.47 (0.26–0.86)		0.47 (0.25–0.87)	
* Salvage surgery				
− (reference)	1	0.290		
+	0.45 (0.11–1.94)			
* Consolidation chemotherapy				
− (reference)	1			
+	0.63 (0.27–1.44)	0.270		
Neutropenia				
Grade 0–2 (reference)	1	0.021	1	0.011
Grade 3–4	3.34 (1.2–9.27)		3.77 (1.35–10.56)	

* Modeled as time-dependent covariates.

**Table 6:** Association between therapy-induced neutropenia and lymphocyte counts in patients with recurrence or residual disease.

Characteristics	Neutropenia		
	Grade 0–2	Grade 3–4	*p*-Value
	(*n* = 7)	(*n* = 31)	
Pretreatment lymphocyte count (cells/μL, mean [sd])	1114 (520)	1325 (451)	0.284
Lymphocyte count at the time of recurrence (residual) disease (cells/μL, mean [sd])	1058 (464)	820 (312)	0.147
Pretreatment to time of recurrence (residual) disease lymphocyte ratio	3.48 (5.93)	0.76 (0.64)	0.012

## Data Availability

The datasets analyzed during the current study are not publicly available but are available from the corresponding author on reasonable request.

## Supplementary Materials

The following supporting information can be downloaded at: https://www.mdpi.com/article/10.3390/cancers18010112/s1, Table S1. Association between therapy-induced neutropenia and 2nd cycle per-agent RDI and myelosuppression management. Figure S1. Kaplan-Meier curves for (a) locoregional control survival and (b) distant metastasis-free survival rate according to neutropenia grade.

## Abbreviations

The following abbreviations are used in this manuscript:

CR	complete response
SD	standard deviation
