# Supplementary material for: Therapy-Induced Neutropenia and Poor Prognosis in Patients with Locally Advanced Esophageal Cancer Who Underwent Concurrent Chemoradiotherapy with Docetaxel, Cisplatin, and 5-Fluorouracil
Source: Cancers (Basel). 2025 Dec 29;18(1):112. doi: 10.3390/cancers18010112 (PMC12785004; doi:10.3390/cancers18010112)
Supplement: Supplementary file 1 [file cancers-18-00112-s001.zip › Supplementary Figure S1.pptx]

## Slide 1
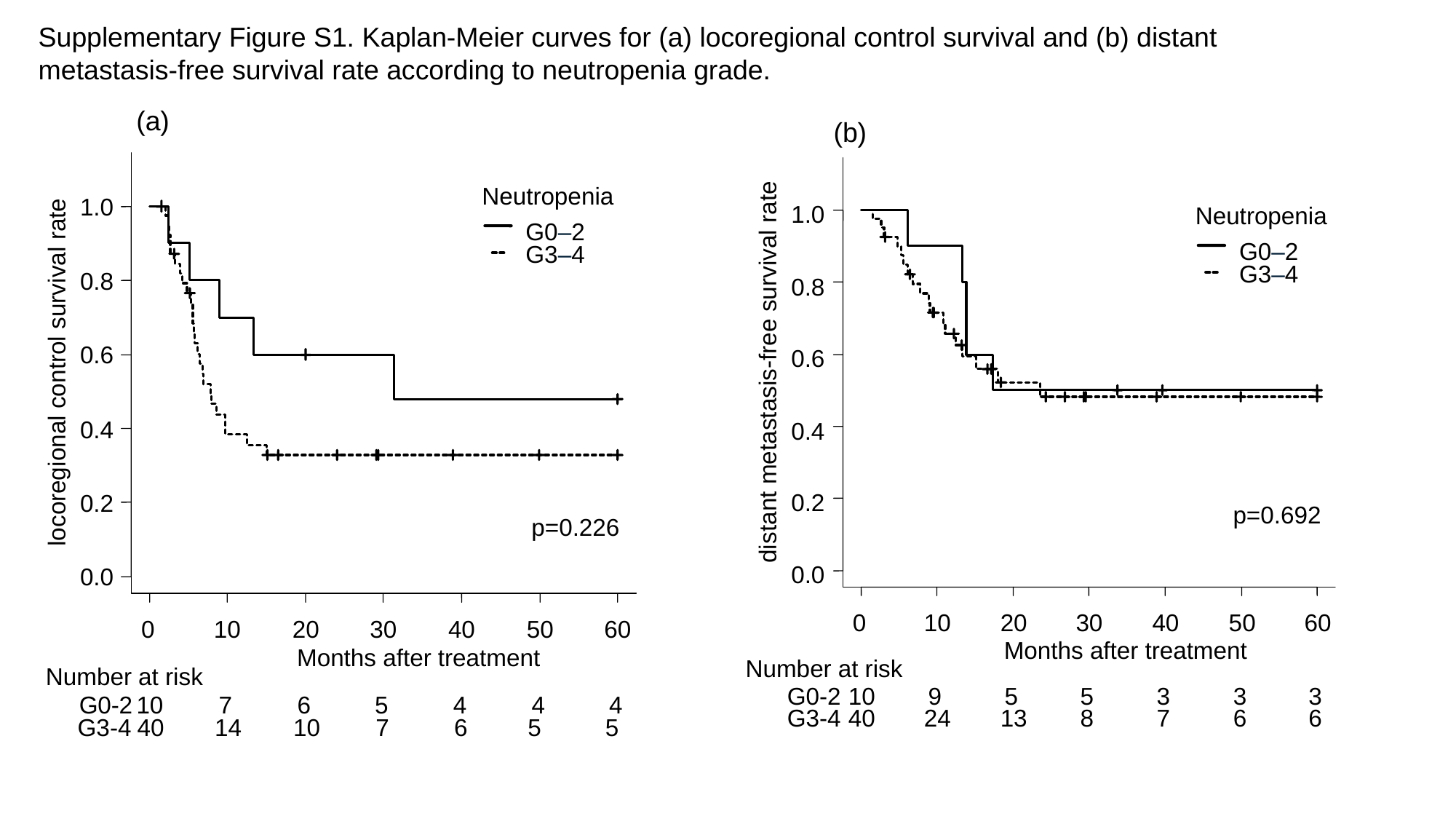

Supplementary Figure S1. Kaplan-Meier curves for (a) locoregional control survival and (b) distant metastasis-free survival rate according to neutropenia grade.
(a)
(b)
Neutropenia
1.0
G0–2
G3–4
0.8
0.6
locoregional control survival rate
0.4
0.2
0.0
0
10
20
30
40
50
60
Months after treatment
Number at risk
G0-2
10
7
6
5
4
4
4
G3-4
40
14
10
7
6
5
5
1.0
Neutropenia
G0–2
G3–4
0.8
0.6
distant metastasis-free survival rate
0.4
0.2
p=0.692
p=0.226
0.0
0
10
20
30
40
50
60
Months after treatment
Number at risk
G0-2
10
9
5
5
3
3
3
G3-4
40
24
13
8
7
6
6
