# Supplementary material for: Therapy-Induced Neutropenia and Poor Prognosis in Patients with Locally Advanced Esophageal Cancer Who Underwent Concurrent Chemoradiotherapy with Docetaxel, Cisplatin, and 5-Fluorouracil
Source: Cancers (Basel). 2025 Dec 29;18(1):112. doi: 10.3390/cancers18010112 (PMC12785004; doi:10.3390/cancers18010112)
Supplement: Supplementary file 1 [file cancers-18-00112-s001.zip › Supplementary Table S1.pdf]

Supplementary Table S1. Association between therapy-induced neutropenia and 2nd cycle per-agent RDI and myelosuppression management.

**Characteristics**	**All patients**	**Neutropenia**		
	(n = 40)	Grade 0–2 (n = 8)	Grade 3–4 (n = 32)	p-value
2nd cycle reduction/omissions				
No reduction/omissions	16 (40.0)	4 (50.0)	12 (37.5)	0.674
Reductions	8 (20.0)	2 (25.0)	14 (43.8)	
Omissions	16 (40.0)	2 (25.0)	6 (18.8)	
2nd cycle RDI for each agent (mean [sd])				
Docetaxel	71.8 (38.2)	68.0 (43.9)	72.7 (37.4)	0.761
CDDP	70.6 (37.5)	68.0 (43.9)	71.3 (36.4)	0.83
5FU	70.9 (37.5)	68.0 (43.9)	71.6 (36.5)	0.814
Reason for reduction/omissions				
Dose reduction due to prior neutropenia	16(69.6)	2 (50.0)	14 (73.7)	0.415
Persistent myelosuppression	4(17.4)	1 (25.0)	3 (15.8)	
Poor general condition	2(8.7)	1 (25.0)	1 (5.3)	
Renal dysfunction after the 1st course	1(4.3)	0 (0.0)	1 (5.3)	
G -CSF usage, No. (%)				
-	2 (4.0)	3 (37.5)	4 (12.5)	0.128
+	48 (96.0)	5 (62.5)	28 (87.5)	
Antibiotics usage, No. (%)				
-	17 (42.5)	5 (62.5)	12 (37.5)	0.27
prophylactic	8 (20.0)	0 (0.0)	8 (25.0)	
Therapeutic	15 (37.5)	3 (37.5)	12 (37.5)	
